# Optimal Design of CFRP Railway Carbody Laminates via Multi-Criterion Fiber Orientation Sensitivity

**DOI:** 10.3390/ma19071355

**Published:** 2026-03-29

**Authors:** Alessio Cascino, Enrico Meli, Andrea Rindi

**Affiliations:** Department of Industrial Engineering, University of Florence, 50139 Florence, Italy; enrico.meli@unifi.it (E.M.); andrea.rindi@unifi.it (A.R.)

**Keywords:** railway vehicle design, laminate optimization, finite element analysis, multilayer composite panels, lightweight design, structural optimization: carbon fiber

## Abstract

The research presented in this work focuses on the structural optimization of a multilayer CFRP (carbon fiber reinforced polymer) laminate integrated within a railway carbody frame. The primary objective is to implement a systematic design methodology aimed at achieving significant mass reduction while preserving the mechanical performance and safety margins required by railway standards. To this end, a multi-stage optimization framework was developed to explore the sensitivity of fiber orientation on the laminate’s failure behavior, directly coupled with high-fidelity finite element models for objective performance extraction. The investigation was initially conducted using an asynchronous optimization strategy, where the orientation of each individual ply was decoupled and analyzed independently. This phase revealed that a tailored, ply-specific approach is essential to address the varying structural requirements across the laminate thickness. Through this methodology, an optimal sequence of 36°/54°/126° was identified, achieving a significant 40.83% reduction in the Tsai–Wu failure index compared to a standard 0°/0°/0° baseline. Subsequently, a synchronous rotation analysis was performed to compare these results against conventional single-orientation design strategies. While the synchronous optimum was identified at 54°, it yielded a lower failure index reduction of 24.81%. The comparison highlights a further 16% performance gain enabled by the asynchronous method. Finally, the validation confirmed that these in-plane improvements were achieved without compromising interlaminar integrity, as the interlaminar shear stress (ILSS) remained constant and safe. This framework provides an objective and rigorous tool for the railway industry, replacing empirical design methods with a high-performance, data-driven approach.

## 1. Introduction

In the contemporary rail sector, the transition toward lightweight architectures is a fundamental requirement to boost energy sustainability and lower operational expenses, especially in high-frequency urban networks. A key strategic shift involves replacing or augmenting conventional metallic frames with advanced composite systems. This approach allows for substantial weight savings while strictly adhering to the demanding safety and durability protocols established by global regulations. In this context, structural optimization has evolved from a specialized technique into a core engineering necessity. Whether in aerospace, automotive, or civil sectors, the application of topology, shape, and sizing methodologies enables a systematic refinement of material distribution. For composite structures, these tools are particularly vital: they allow designers to maximize the inherent benefits of the material, ensuring that high performance and resource efficiency are achieved simultaneously through precise structural tailoring. While the aerospace industry has historically pioneered the use of composite materials to achieve extreme performance thresholds [1,2,3], a comparable technological shift is now becoming mandatory for the railway sector. In aeronautics, the adoption of specialized hybrid systems, such as fiber metal laminates (FMLs), has become standard for fuselage assembly and critical structural panels. These solutions are primarily chosen for their exceptional fatigue resistance and their capacity for energy dissipation during impact [4,5,6]. Following a similar trajectory, the automotive sector has integrated these advanced systems into both primary load-carrying frames and secondary parts, including bodywork and internal cabin modules. This transition is essential for producing lightweight, high-efficiency vehicles that maintain high standards of crashworthiness and structural longevity [7,8,9,10]. Additionally, the marine industry capitalizes on the inherent chemical and environmental stability of composites, employing them for hulls and superstructures that must endure the highly corrosive conditions of maritime environments [11,12,13]. This broad success across diverse engineering fields provides a solid foundation for the strategic implementation of similar lightweight methodologies within modern railway carbody design. The implementation of innovative materials coupled with advanced structural optimization has recently yielded encouraging outcomes in the rail industry. Research has increasingly focused on the mechanical validation and refinement of composite and sandwich configurations for carbody applications. Through finite element analysis (FEA), studies have demonstrated the potential for strong weight savings [14], while experimental testing on curved glass fiber reinforced polymer (GFRP) structures has verified their impact resilience and operational viability for rolling stock [15]. Current optimization trends have expanded to include topology, shape, and sizing adjustments for internal ribs and external shells [16]. Despite this progress, aluminum alloys remain the benchmark for modern carbody construction, favored for their established balance of specific strength, corrosion protection, and ease of industrial processing [17]. Nevertheless, the emergence of carbon fiber reinforced polymers (CFRPs) has redefined the limits of weight optimization. Contemporary research is now focused on benchmarking CFRPs against aluminum, specifically evaluating their mechanical efficiency, fatigue life, and cost-effectiveness to justify their large-scale adoption in future railway generations [18,19,20,21,22]. The mechanical performance of reinforced polymers is intrinsically linked to the spatial arrangement of the fibers, as the inherent anisotropy of these materials allows for high structural efficiency only when the reinforcement is aligned with the principal stress fields. Recent studies on jute-reinforced systems emphasize that tailoring the reinforcement direction is the primary driver for optimizing the stiffness-to-weight ratio in natural composites [23]. In more traditional engineering applications, the influence of fiber orientation has proven critical even for cementitious composites, where the specific loading condition dictates the required reinforcement layout to ensure structural integrity [24]. The challenge of ensuring precise fiber alignment is also a major focus of manufacturing research. Advanced characterization techniques, such as structure tensor analysis, have been employed to quantify the deviations introduced by different production methods in unidirectional composites [25]. Furthermore, innovative processing techniques, like convergent die-induced orientation, demonstrate how controlling the local fiber architecture during fabrication can enhance the mechanical consistency of the final component [26]. The sensitivity of tensile properties in carbon fiber/epoxy laminates further validates these findings, confirming that even minor angular deviations can significantly degrade the load-carrying capacity [27]. Recent advancements in additive manufacturing, specifically nozzle-impregnation 3D printing, have further expanded the ability to customize fiber paths for enhanced performance [28]. Beyond pure mechanical strength, the specific orientation of the layers has been shown to directly affect the health monitoring capabilities and damage detection signals in multi-layered systems [29]. Comprehensive reviews of CFRP composites reinforce this, showing that mechanical properties vary drastically across different fiber configurations [30]. From a numerical perspective, advanced approaches now combine topology optimization with level-set methods to simultaneously refine shape and fiber direction [31]. While finite element modeling remains a reliable tool for predicting the behavior of natural fiber composites under various orientations [32], the most recent developments focus on optimizing failure characteristics. Specifically, research into laminated structures suggests that identifying the ideal stacking sequence is essential for controlling damage evolution and maximizing the structural reserve of the system [33]. Despite their benefits, the integration of composite laminates faces complex challenges, including sophisticated failure modes like matrix cracking and interlaminar separation, alongside high production costs and the need for dedicated maintenance cycles. To overcome these barriers, contemporary research is focused on improving manufacturing scalability, investigating eco-friendly hybrid or bio-composites, and refining structural health monitoring (SHM) systems through advanced computational logic [34,35,36,37]. Beyond the carbody shell, lightweight design strategies are being extended to other vital components, such as bolster beams and bogie frames. In these high-demand assemblies, the combination of specialized material selection and structural optimization has successfully reduced mass while ensuring strict adherence to the EN 13749 safety standards [38,39,40,41,42,43]. The drive toward energy-efficient rail transport has established finite element analysis (FEA) as a prerequisite for validating structural integrity under operational loads. Recent studies highlight the effectiveness of this methodology: engineered composite architectures have achieved weight reductions of 28% compared to aluminum, without sacrificing impact or fatigue performance [44]. Even more significant gains have been reported with pultruded GFRP panels, which reached a 35.5% mass saving over traditional steel structures [45]. Within this framework, the real-time monitoring of rolling stock provides the empirical data necessary to calibrate increasingly precise optimization models for both passenger and freight applications [46,47,48,49,50,51]. Collectively, these milestones demonstrate that advanced numerical simulation is the indispensable catalyst for the transition from conventional metallic carbodies to high-performance composite systems. In conclusion, the state-of-the-art demonstrates that while multilayered composites possess extraordinary potential for the rail industry due to their exceptional specific stiffness and design versatility, their industrial application frequently relies on standard or sub-optimal stacking sequences. A systematic approach to ply-by-ply orientation remains largely underutilized in practical design workflows. Addressing this gap, the current study proposes a novel, high-speed optimization framework specifically engineered for railway carbody components, yet versatile enough for broader structural applications. By integrating a fully automated computational pipeline with an intuitive graphical interface for result interpretation, the methodology allows for the rapid identification of the ideal fiber orientation. The proposed procedure effectively balances regulatory safety margins with peak structural efficiency. Ultimately, this research provides a streamlined, performance-oriented strategy that facilitates the transition from empirical design to a more rigorous, optimized exploitation of composite materials in modern railway engineering.

## 2. Methodology

Composite multilayer materials are increasingly gaining prominence in modern structural design due to their exceptional specific stiffness and the unique ability to tailor directional properties to specific load paths. Unlike traditional isotropic materials, the performance of carbon fiber-reinforced polymers (CFRPs) is intrinsically linked to the angular orientation of the individual ply. However, identifying the optimal fiber orientation often remains a challenge in industrial practice, frequently relying on simplified rules of thumb rather than systematic numerical evidence. This gap can lead to sub-optimal designs where the full lightweight potential of the composite is not exploited, or where interlaminar risks are overlooked. In order to address this challenge, the methodology developed in this work provides a structured framework designed to assess the sensitivity of laminate performance through two distinct optimization strategies: asynchronous ply-by-ply sensitivity and synchronous global orientation. To ensure a balance between numerical accuracy and computational efficiency during these procedures, the laminate is modeled using a “cluster” approach. Specifically, each orientation layer is assigned a nominal thickness of 1 mm, acting as a functional structural block. While physical prepreg plies are typically thinner (0.12–0.18 mm), these 1 mm clusters serve as the primary design variables for the optimization engine. This choice is justified by the large scale of the railway underframe, where the structural impact of a single 0.15 mm lamina is negligible compared to the global stiffness and the magnitude of the applied loads. This strategy significantly reduces the search space complexity while maintaining strict adherence to the structural response required by reference standard. The strength of the proposed approach lies in its high level of computational automation, which couples finite element analysis (FEA) with an iterative post-processing engine to evaluate structural integrity through a multi-criterion approach. The methodology is organized into five main phases:

(1) The process begins with the development of a high-fidelity finite element (FE) model of the component. This stage involves the accurate definition of geometry, mesh discretization, boundary conditions, and operational loading scenarios. The FE model serves as the baseline for all subsequent sensitivity iterations, ensuring that the stress distributions used for optimization reflect the actual service conditions of the structure.
(2) The second step involves defining the mechanical properties of the CFRP constituent layers. Each ply is characterized by its orthotropic elastic constants and its strength parameters, expressed in MPa to ensure consistency with the FE solver output. These properties are integrated into the FE environment using traditional shell theory formulations, allowing for a detailed representation of the stress tensors at each ply interface and mid-plane.
(3) The core of the methodology is an automated loop that executes a massive sweep of simulations across a full 180° rotation spectrum. This framework is designed to handle two separate study cases:
  (a) Asynchronous strategy (ply-by-ply): the orientation of a single target ply is varied while keeping the rest of the stack constant. This identifies the specific contribution and optimal alignment of each individual layer within the laminate.
  (b) Synchronous strategy (global rotation): all plies within the laminate are rotated simultaneously by the same angle. This strategy evaluates the global directional sensitivity of the material, effectively treating the laminate as a single orthotropic block to identify the primary principal load directions.
(4) In this phase, the data extracted from the iterative simulations are processed through a dual-criterion evaluation engine. To ensure a comprehensive assessment of structural integrity, the methodology accounts for both in-plane and out-of-plane failure modes:
  (a) In-plane failure: evaluated using the Tsai–Wu failure criterion, which accounts for the interaction between different stress components within the fiber–matrix system.
  (b) Interlaminar failure: assessed via the interlaminar shear stress (ILSS) utilization ratio. This is particularly critical in the asynchronous strategy, where changing the relative angle between plies can significantly alter the shear transfer at the interfaces.
(5) The final step identifies the optimal configuration by calculating a combined criticality index. This index merges the normalized envelopes of the Tsai–Wu and ILSS criteria using a weighted approach. A comparative analysis is then performed between the asynchronous and synchronous results. This comparison allows for a scientific validation of the benefits of a differentiated layup versus a simpler aligned configuration, providing a definitive optimization table and performance maps that guide the final design choice toward maximum structural efficiency.

By integrating these dual optimization strategies within a unified computational workflow, the methodology establishes a rigorous basis for evaluating the trade-offs between local ply performance and global structural response. This structured approach aims to bridge the long-standing gap in composite material design, where the lack of systematic orientation studies often leads to conservative or sub-optimal configurations, providing instead a clear path toward high-performance, tailored structural solutions.

## 3. Railway Carbody

**Figure 1:**
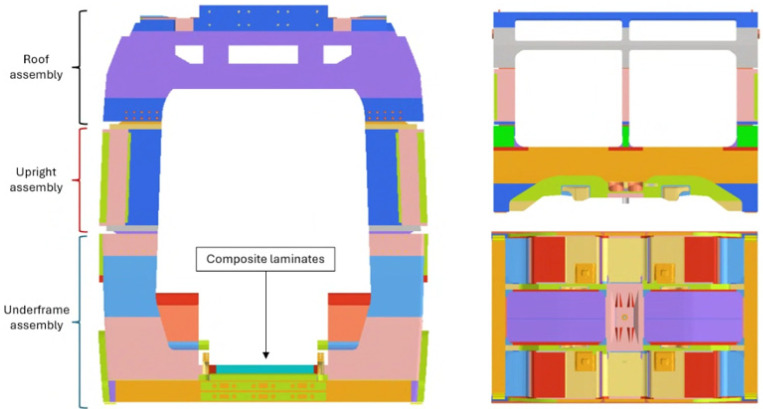
FE model of the carbody structure, global view.

The vehicle analyzed in this research is a modern, low-floor light rail unit, typically composed of five or more modules depending on the specific service requirements. Designed for urban transport, this tramway architecture introduces specific structural challenges due to its distinctive design requirements. Regarding the carbody modules, which are the central focus of this study, two main configurations exist. The first type is a compact unit that interfaces directly with the bogies and is designed to house various underframe subsystems. The second type is an elongated module, not connected to the bogies, which provides the space required for passenger doors and access. The finite element (FE) model of the first type carbody, which is the object of this research, and all the main structural carbody assemblies are illustrated in Figure 1.

**Figure 2:**
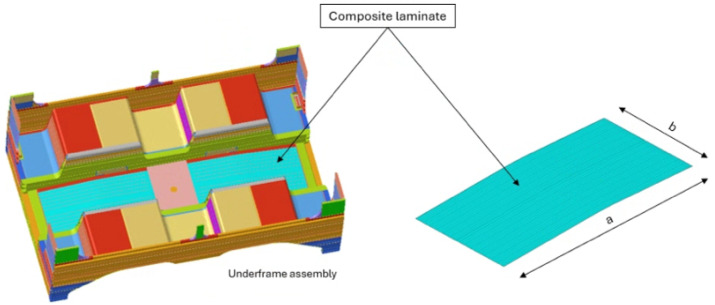
Composite laminates positioned on the underframe assembly of the carbody structure (FE model).

Specifically, the adoption of a low floor requires a substantial reconfiguration of the underframe, leading to complex load conditions throughout the assembly. In order to maintain structural integrity, reinforcements are strategically placed in critical areas, such as the articulation joints and door openings. Furthermore, the specific layout necessitates alternative solutions for housing vital systems and various equipment, which are frequently integrated within the bogies or repositioned on the roof. These technical measures are essential to balance structural strength with passenger capacity and accessibility regulations. The original construction of the vehicle utilizes aluminum alloy for the main carbody structures, while construction steel is used for the bogie frames and composite materials for the driver’s cabins. The carbody assembly is primarily composed of extruded aluminum profiles, joined through welding processes. The specific material utilized for these structural elements is the EN AW 6106 T6 aluminum alloy, as specified by the EN 1999-1-1:2014 standard [52]. This 6xxx series alloy, characterized by magnesium and silicon as primary alloying agents, provides an optimal combination of mechanical strength, weldability, and corrosion resistance, essential properties for lightweight railway applications. The T6 temper, resulting from solution heat treatment followed by artificial aging, ensures high yield and tensile strength while preserving the formability and fatigue endurance necessary for urban transit service. In a move toward a hybrid structural architecture, multilayer composite laminates were integrated into specific zones of the aluminum frame, as shown in Figure 2. Each laminate panel features nominal dimensions of 1400 mm in length (a) and 750 mm in width (b). While several areas, including the roof, side walls, and internal supports, were identified as suitable for composite application, this research specifically targets the underframe. In this study, the original aluminum underframe component was replaced with tailored CFRP composite laminates to evaluate its potential for weight optimization and structural efficiency. In order to fully exploit the anisotropic nature of the material, a specific angular optimization methodology was developed to determine the most effective stacking sequence for the given load paths. This component is subjected to significant bending loads, a stress state where the high specific stiffness of composite materials, when combined with optimized fiber orientations, offers the most substantial advantages for mass reduction without compromising safety.

### 3.1. CFRP Composite Laminates

The integration of composite laminates into modern transportation structures is driven by their optimal specific mechanical properties and inherent resistance to environmental degradation. The primary advantage of these materials is the high degree of design freedom they offer, allowing for the precise calibration of the structural response. By assembling multiple layers, or plies, engineers can manipulate the global behavior of the laminate through the selection of specific fiber orientations, layer thicknesses, and stacking sequences. A standard laminate assembly typically comprises three or more plies, where the collective mechanical performance is a function of the individual ply properties and their mutual interaction. This anisotropic nature enables the development of components specifically tailored to resist the primary load paths of the application. Consequently, the use of CFRP allows for a sophisticated trade-off between mass optimization, structural integrity, and manufacturing constraints, making it possible to achieve performance levels that exceed those of conventional isotropic materials under complex loading scenarios. The primary aim of this study is the design and structural optimization of a tramway carbody, with the main investigation centered on the configuration of the composite laminate. Specifically, the research focuses on the optimization of fiber orientation as the key design variable. The study aims to demonstrate how the proposed methodology is able to optimize the orientation fiber sequence and improve the structural behavior of the laminates. The research activity adopted a CFRP laminate composed of three plies. The mechanical properties of each ply are summarized in Table 1.

### 3.2. FE Model Description and Simulation Settings

**Figure 3:**
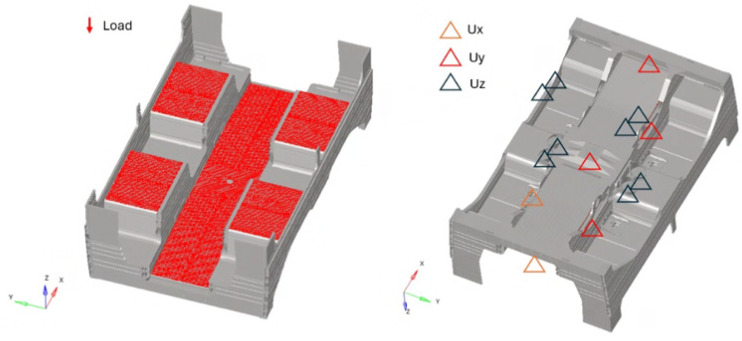
FE model: load and constraint conditions.

The structural response was evaluated using a high-fidelity finite element (FE) model consisting of nearly 1 million nodes, created using Altair Hypermesh environment. The entire carbody and its sub-components were discretized using first-order QUAD4 shell elements. While a consistent 2D meshing approach was applied to both metallic and composite parts, the laminate elements were assigned specialized properties to account for the individual ply characteristics. To streamline the optimization process and prevent computational redundancy during permutational analyses, a dedicated local subdomain was established for the composite region. This approach avoided the introduction of contact interfaces, thereby reducing the computational cost of the analysis. The discretization was based on an average element size of 18 mm. This value was determined through a rigorous mesh sensitivity study, which explored a range from 25 mm down to 14 mm. The analysis confirmed that stress and displacement values stabilized at 18 mm, ensuring a balance between computational efficiency and result reliability. Connections such as rivets were simulated using a combination of RBE2 rigid elements and 1D beam elements, while onboard equipment was represented by localized masses coupled to the structure via RBE3 elements to ensure realistic load distribution. Structural interfaces, particularly the bonded joints between the laminate and the aluminum frame, were modelled using a “FREEZE” contact formulation. This method ensures linear load transmission across the connected nodes, maintaining model stability for the optimization phase. The vehicle is classified under the P-V category (Light-rail/Passenger vehicles) according to the EN 12663:2024 standard [54], and all mass assessments followed the EN 15663:2019 standard [55]. This includes the evaluation of tare mass (C0), operational mass (C2), and maximum laden mass (C4). The numerical simulations were conducted on a workstation featuring an Intel(R) Xeon(R) CPU E5-2643 v4 @ 3.40 GHz with 32 GB of RAM. The primary load case selected for the optimization is the most critical for the underframe region: the maximum vertical load (C4) increased by a 30% dynamic factor. This scenario assumes a high-density passenger load of six people per square meter (approx. 70 kg per person), representing the most demanding condition for the structural validation of the composite panel. Boundary conditions were established to replicate an isostatic constraint configuration consistent with real in-service operational states. Vertical degrees of freedom were restricted at the secondary suspension seats, while lateral displacements were constrained at the side-pad interfaces to ensure lateral stability. To prevent rigid-body motion along the track direction, longitudinal constraints were applied directly to the structural end-face of the carbody. This setup ensures that the reaction forces are distributed according to the real load paths of the vehicle frame. Figure 3 clearly shows the load and constraint conditions. Finally, to evaluate the practical efficiency of the proposed multi-stage framework, the computational time was monitored considering the adoption of a high-fidelity FE model of the complete railway carbody, accounting for the full structural complexity and standardized load cases. A typical optimization cycle reaches convergence for a specific design target in approximately 7 h. This timeframe allows for a rapid quantitative assessment of the structure while maintaining strict adherence to the international load conditions defined by the EN 12663:2024 standard. The ability to transition from a baseline configuration to a fully optimized, load-specific layup in a matter of hours—rather than the several days typically required by the empirical ‘rules of thumb’ and manual trial-and-error procedures still prevalent in the railway sector—demonstrates the framework’s suitability for the iterative design loops of the industrial prototyping phase.

### 3.3. Failure Criteria and Optimization Strategy

With the aim of evaluating the structural integrity of the composite laminate under the complex loading conditions of the carbody, a multi-criterion approach was adopted. This methodology combines the assessment of in-plane ply failure with the analysis of interlaminar shear stability. The reference failure parameters for all plies are reported in Table 2.

#### 3.3.1. Tsai–Wu Failure Criterion

The in-plane failure resistance was assessed using the Tsai–Wu phenomenological criterion, which accounts for the interaction between different stress components. For a plane stress state, the failure index $FI_{Tsai-Wu}$ is expressed as:

(1) $$FI_{Tsai-Wu}=F_{1}\sigma_{1}+F_{2}\sigma_{2}+F_{11}{\sigma_{1}}^{2}+F_{22}{\sigma_{2}}^{2}+F_{66}{\tau_{12}}^{2}+2F_{12}\sigma_{1}\sigma_{2}$$

where $\sigma_{1},\sigma_{2}$ are the normal stresses along the material directions and $\tau_{12}$ is the in-plane shear stress. The terms $X_{t},X_{c},Y_{t},Y_{c},S$ denote the corresponding tensile, compressive, and shear strengths. The main coefficients are calculated as $F_{1}=\frac{1}{X_{t}}-\frac{1}{X_{c}}$, $F_{2}=\frac{1}{Y_{t}}-\frac{1}{Y_{c}}$, $F_{11}=\frac{1}{X_{t}X_{c}}$, $F_{22}=\frac{1}{Y_{t}Y_{c}}$, $F_{66}=\frac{1}{S^{2}}$, and $F_{12}=-0.5\sqrt{F_{11}F_{22}}$. Finally, a value of $FI_{Tsai-Wu}\;<\;1$ indicates that the ply is structurally safe, while $FI_{Tsai-Wu}\;\geq \;1$ predicts failure.

#### 3.3.2. Interlaminar Shear Stress (ILSS)

To prevent delamination, the out-of-plane performance was monitored through the interlaminar shear stress (ILSS). The combined interlaminar stress was calculated using the transverse shear components $\tau_{1z}$ and $\tau_{2z}$ extracted from the FE model:

(2) $$ILSS\;=\;\sqrt{\tau_{1z}^{2}+\tau_{2z}^{2}}$$

This value was compared against the material’s interlaminar shear limit $\tau_{IL}$ to ensure that the optimization of fiber angles did not introduce secondary failure modes between the layers.

#### 3.3.3. Design Envelope and Multi-Objective Weighting

The search for the optimal stacking sequence was conducted by constructing a design envelope. Since the structure consists of multiple elements, the envelope considers the maximum (worst-case) failure indices across the entire critical area for each orientation angle α. To balance the requirements of in-plane strength and interlaminar stability, a combined performance index ($I_{C}\left(\alpha \right)$) was formulated. Both the Tsai–Wu index and the ILSS values were normalized to their respective maximum observed values during the sensitivity analysis to allow for a direct comparison:

(3) $$I_{C}\left(\alpha \right)=w_{TW}\cdot \left(\frac{FI_{TW}\left(\alpha \right)}{FI_{TW,max}}\right)+w_{ILSS}\cdot \left(\frac{ILSS\left(\alpha \right)}{ILSS_{max}}\right)$$

where $w_{TW}$ and $w_{ILSS}$ are the weighting factors. For this study, an equal weighting of 0.5 was adopted to ensure that the global failure index and the local stress concentrations were granted equivalent structural significance. This balanced approach is justified by the requirement to mitigate both the risk of onset failure and the progression of damage with equal priority during the initial design phase. Consequently, the optimal orientation for each ply was identified by determining the value of α that minimizes the combined index $I_{C}\left(\alpha \right)$.

## 4. Results and Discussion

The following section presents a detailed analysis of the results obtained from the automated sensitivity framework, evaluating the structural response of the composite laminate under both asynchronous and synchronous rotation strategies. Although the current study focused on a high-performance carbon/epoxy system as described in Table 1 and Table 2, to demonstrate the benefits of weight reduction in low-floor railway architectures, the proposed multi-stage optimization framework is inherently material-agnostic. The mathematical engine of the algorithm operates on the stiffness matrix, which is constructed based on the material and laminate characteristics appropriately defined within the finite element (FE) model. This ensures that the methodology can be seamlessly adapted to any fiber/resin combination. The same concept was applied for failure criteria. For instance, by simply updating the input mechanical properties and the corresponding failure envelopes, the framework can optimize glass fiber reinforced polymers (GFRPs) for cost-sensitive components or hybrid carbon–glass architectures for improved impact resistance. The core logic of the discrete ply-level optimization remains unchanged, regardless of the specific material constants used. This versatility ensures that the tool can be deployed across a wide range of rolling stock applications, from secondary structural fairings to primary load-bearing chassis. The discussion is structured to provide a comprehensive understanding of the laminate’s behavior, starting with the asynchronous ply-by-ply analysis, which highlights the individual contribution of each layer to the overall failure indices. Subsequently, the synchronous global sensitivity results are examined to identify the primary load paths of the structure when treated as a unified orthotropic system. By comparing these two approaches, the trade-offs between local ply optimization and global interlaminar stability are critically discussed. Special emphasis is placed on the interaction between the Tsai–Wu failure criterion and the interlaminar shear stress (ILSS), identifying not only the optimal fiber orientations for maximum strength but also the robustness of the design against delamination. This dual-layered analysis serves to validate the proposed methodology as a superior alternative to conventional, non-systematic design practices. Finally, a global sensitivity comparing the results of the different approaches is discussed.

### 4.1. Fiber Orientation Sensitivity Framework

**Figure 4:**
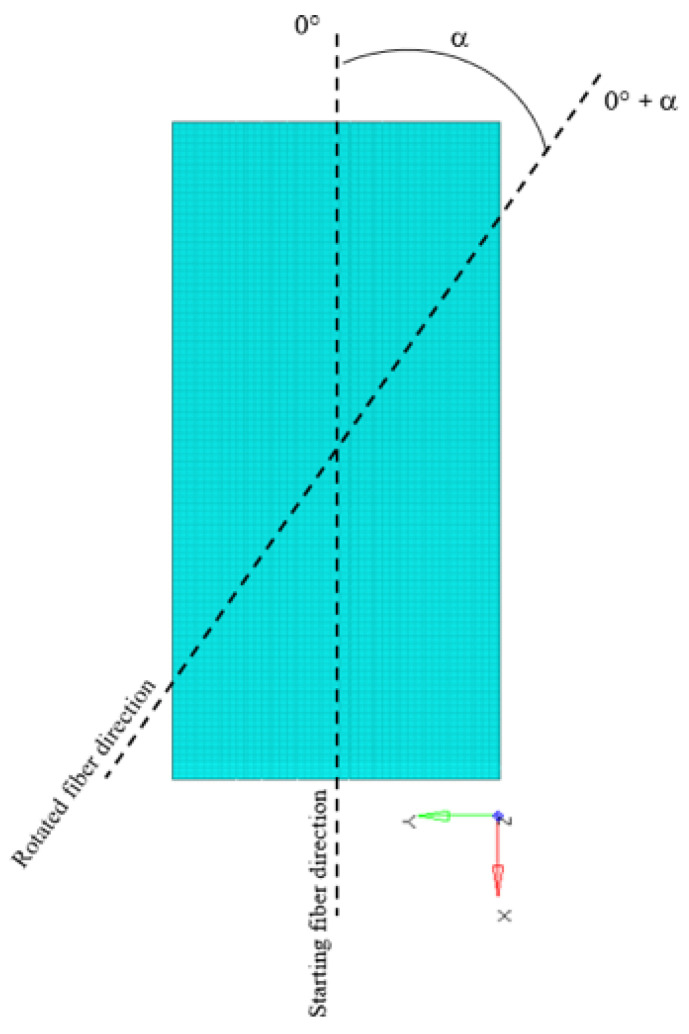
Fiber angular rotation scheme.

The results of the automated sensitivity analysis provide a comprehensive map of the structural response as a function of fiber orientation. To capture the full orthotropic behavior of the carbon fiber laminate, a rotation spectrum of 0° to 171° was selected, utilizing a resolution of 9°. This angular increment was chosen as a balanced trade-off between computational cost and the granularity required to identify sharp gradients in the failure indices. It is important to note that the study was bounded at 180° due to the inherent orthotropic symmetry of carbon fibers; since the fibers possess directional properties but lack a specific “head” or “tail” (non-polar symmetry), an orientation of α is physically equivalent to α + 180°. Consequently, the 20 iterations performed for each study case sufficiently covered the entire unique design space for the laminate. Figure 4 describes the fiber angular rotation.

### 4.2. Asynchronous Analysis: Individual Ply Sensitivity

**Figure 5:**
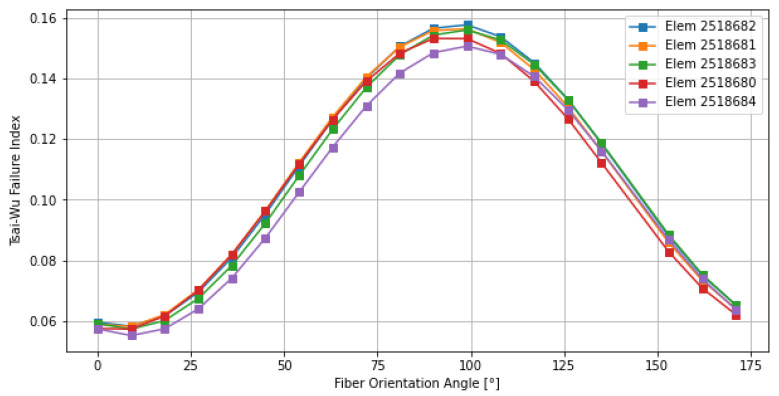
Sensitivity of the Tsai–Wu failure index for the most critical elements of Ply 1.

The first phase of the investigation focuses on the asynchronous strategy, where each layer is analyzed independently to determine its specific influence on the global failure envelope. In order to ensure the structural integrity of the entire component, the optimization engine identifies and tracks only the most critical elements, specifically those exhibiting the highest peak values of the Tsai–Wu failure index. By focusing the discussion on these high-stress “hotspots”, the methodology guarantees a conservative and safety-oriented design approach: if the fiber orientation is optimized for the elements under the most severe loading conditions, the safety margin for the rest of the structure is inherently maximized. As shown in Figure 5, Ply 1 exhibits a high sensitivity to angular variation. The transition from the optimal 36° to the critical 100° zone results in a nearly 170% increase in the Tsai–Wu failure index. This sharp gradient suggests that the outer layer is primarily subjected to a well-defined uniaxial tension–compression field. The optimal orientation at 36° effectively aligns the high-modulus carbon fibers with these principal stress trajectories. The narrowness of the “valley” in the graph indicates that even a small deviation (±15°) from the optimal angle could significantly compromise the structural integrity of the external skin, making precision in the manufacturing phase crucial for this specific layer.

**Figure 6:**
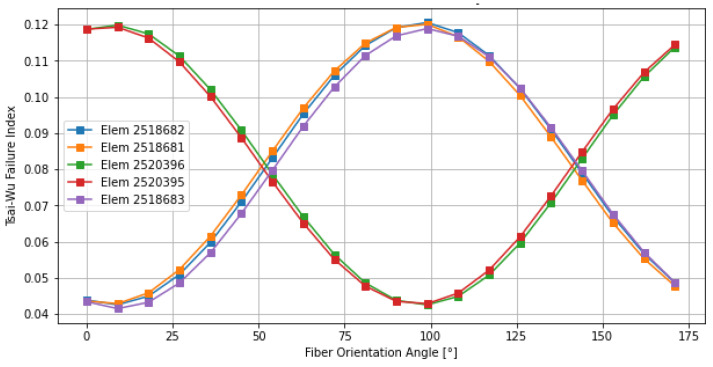
Sensitivity of the Tsai–Wu failure index for the most critical elements of Ply 2.

The behavior of Ply 2, illustrated in Figure 6, is characterized by a “W-shaped” profile. Unlike the clean parabolic trend of Ply 1, Ply 2 showed two regions of relative stability. However, the global minimum at 54° provided the most robust configuration. The presence of multiple local minima suggests that this layer acts as a structural buffer, where the stress state is less dominated by a single load path and more influenced by the interaction between adjacent plies. The shift from 36° (Ply 1) to 54° (Ply 2) confirmed a significant reorientation of the principal loading paths through the laminate thickness., a phenomenon that standard [0/45/90] layups could fail to capture.

**Figure 7:**
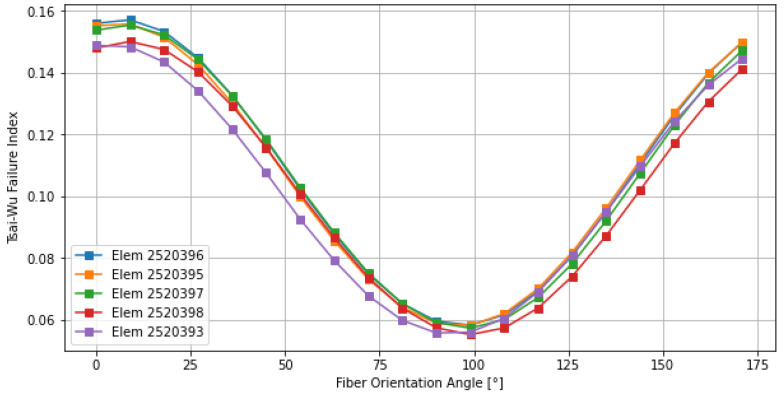
Sensitivity of the Tsai–Wu failure index for the most critical elements of Ply 3.

Ply 3 (Figure 7) found its optimum at 126° (equivalent to −54°). This orientation was nearly orthogonal to the optimal angle of the first layer, suggesting a fundamental role in balancing the shear components of the laminate. In railway carbody structures, the combination of longitudinal, lateral, and torsional loads creates a complex internal stress field. Because these forces act in different directions, the optimal fiber orientation must change through the thickness of the laminate to effectively support the resulting load distribution. The optimization of Ply 3 at 126° demonstrates the methodology’s ability to “deploy” fibers specifically to counteract these secondary but critical stress components, ensuring that the internal face of the laminate is as protected as the external one.

### 4.3. Multi-Criterion Optimization: The Combined Envelope Approach

**Figure 8:**
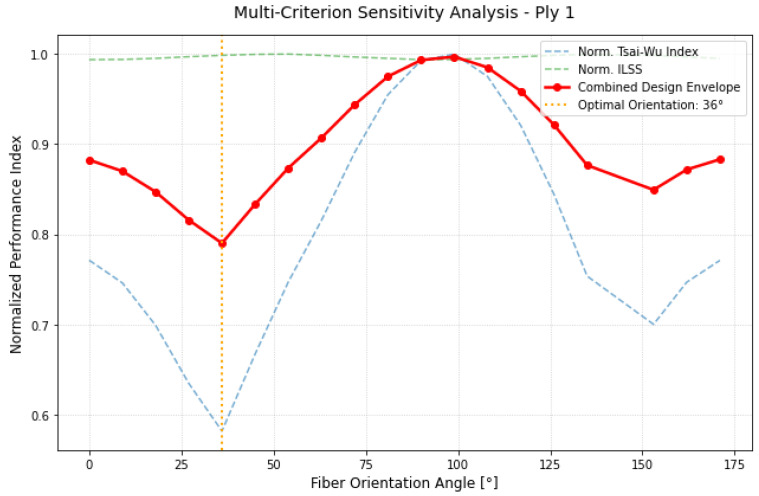
Multi-criterion sensitivity map for Ply 1.

The implementation of the multi-criterion envelope provides a detailed visualization of the laminate’s sensitivity to fiber orientation. By monitoring the combined performance index ($I_{C}\left(\alpha \right)$), it is possible to observe how the competition between in-plane resistance and interlaminar stability determines the final design. As shown in Figure 8, Ply 1 exhibited the highest sensitivity to angular variation, confirming its role as the primary structural skin of the underframe. The Tsai–Wu failure index (blue dashed line) displayed a pronounced parabolic trend, reaching its minimum at approximately 36°. This steep gradient highlights a state of high-magnitude membrane stress; any significant deviation from this optimal angle (for instance, ±10°) would shift the load-bearing requirement from the fibers to the resin matrix, rapidly increasing the risk of structural failure. In contrast, the normalized interlaminar shear stress (ILSS), represented by the green dashed line, remained remarkably stable across the entire 180° rotation spectrum. This indicates that for the external layer, the shear stress transfer at the Ply 1–Ply 2 interface is not the critical design constraint. As a result, the combined red envelope ($I_{C}\left(\alpha \right)$) was almost entirely governed by the in-plane performance of the material. The analysis identified 36° as the clear global optimum, ensuring the maximum structural reserve for the outer reinforcement while maintaining a balanced interaction with the underlying layers.

**Figure 9:**
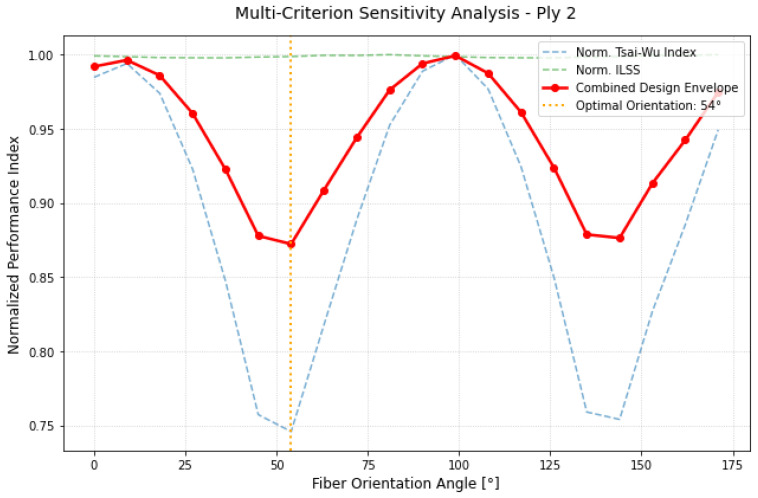
Multi-criterion sensitivity map for Ply 2.

The analysis of the intermediate layer, described in Figure 9, highlights a significant transition in the internal stress distribution. Unlike the outer skin, the Tsai–Wu curve for Ply 2 exhibited a broader “stability valley”, suggesting a reduced sensitivity to minor angular misalignments. This behavior points to a more complex, multi-axial stress state, where the mechanical demand is shared more broadly across the reinforcement. By processing the interaction between in-plane and transverse shear components, the optimization algorithm identified the absolute minimum of the combined performance index ($I_{C}\left(\alpha \right)$.) at 54°. The plateau observed in the $I_{C}\left(\alpha \right)$ curve between 40° and 70° (a higher number of measurement angles would be improved its view) indicates a robust structural response, where the layer maintains low criticality across a wide angular range. This “buffer” effect confirms that Ply 2 acts as a structural bridge within the stacking sequence; however, the 54° set-point remains the configuration of maximum efficiency, where the alignment with the local load paths minimizes the overall failure risk for the internal core.

**Figure 10:**
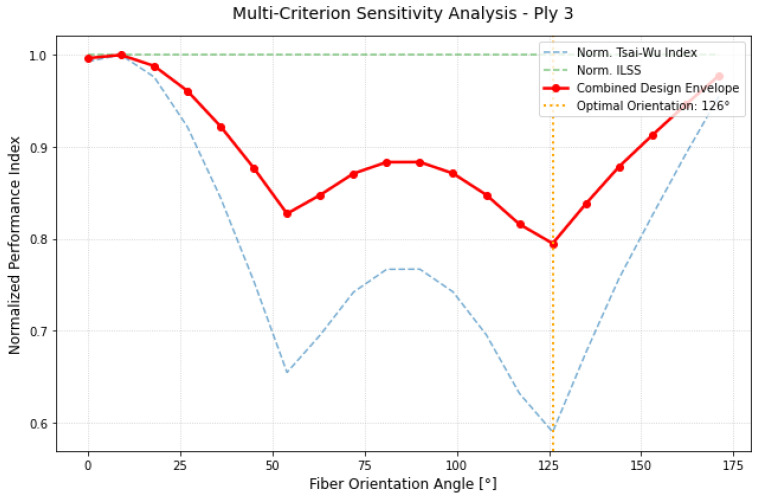
Multi-criterion sensitivity map for Ply 3.

The results for Ply 3, reported in Figure 10, underscore the strategic necessity of the asynchronous optimization approach. Ply 3 exhibited a global minimum at 126° (equivalent to −54°), revealing a significant angular shift compared to the outer layers. This 90° displacement relative to the Ply 1 optimum clearly demonstrates how the principal loading directions vary as a function of depth within the laminate thickness. Under a traditional, uniform orientation strategy, this layer would have been forced to operate in a highly sub-optimal regime, particularly near the 40° mark where its failure index reaches its peak. By decoupling the design variables, the combined performance index $I_{C}\left(\alpha \right)$ successfully identified the specific orientation required to balance the internal load distribution. The red envelope confirms that this tailored, non-standard layup is essential for structural integrity; it ensures that the innermost reinforcement contributes effectively to the total stiffness while maintaining safety margins that would be unachievable with a conventional balanced sequence.

### 4.4. Synchronous Global Sensitivity Analysis

**Figure 11:**
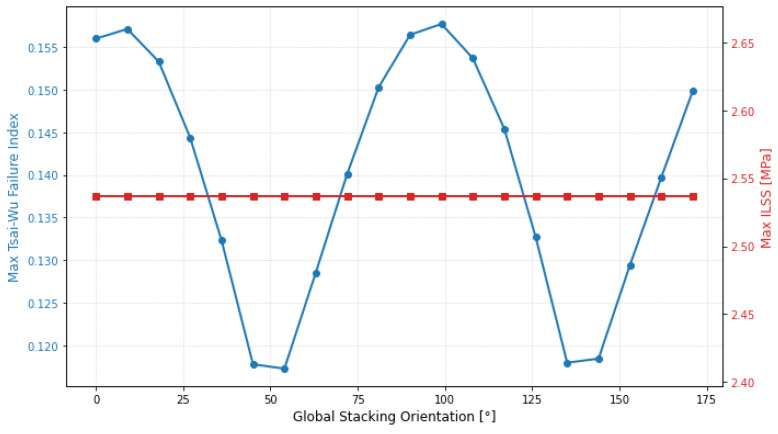
Global sensitivity map for synchronous ply rotation.

While the previous sections focused on the individual contribution of each ply, the synchronous strategy evaluates the structural response when the entire laminate is rotated as a unified orthotropic entity. This study is vital for identifying the primary principal load paths of the component and assessing the global stability of the stacking sequence. As illustrated in Figure 11, the global Tsai–Wu index (blue line) exhibited a distinct periodic behavior with two primary minimums.

The absolute minimum for the entire laminate was identified at 54° (and its symmetrical counterpart near 144°), where the failure index dropped to its lowest point (approx. 0.117). This confirms that the 54° direction represents the dominant load path for the structure, as also observed in the asynchronous study. Conversely, orientations near 10° and 100° represent the most critical configurations, where the failure index peaks at 0.158. In these regions, the laminate’s global stiffness is poorly aligned with the external force flux, significantly reducing the safety margin. One of the most remarkable finding in Figure 11 was the trend of the interlaminar shear stress (ILSS), represented by the green line. Throughout the entire 180° rotation, the ILSS remained perfectly constant at 2.53 MPa. This invariance is a direct consequence of the synchronous rotation. Since all plies rotate together, the relative angle between adjacent layers remains unchanged. This preserves the shear transfer mechanisms at the interfaces, keeping the interlaminar demand static regardless of the global orientation. The fact that 2.53 MPa is a very low value for standard carbon fiber resins confirms that the structure is inherently “delamination-proof” under these loading conditions. This provides the designer with the freedom to optimize the fiber orientation based solely on the in-plane Tsai–Wu criteria without worrying about interlaminar failure.

### 4.5. Global Sensitivity Analysis, Final Comparison, and Real Test Strategy

The final stage of the research involves a direct comparison between the identified optimal configurations and a standard baseline. This validation is essential to quantify the structural benefits of the proposed asynchronous methodology. The results of the three high-fidelity FE simulations are summarized in Table 3.

**Figure 12:**
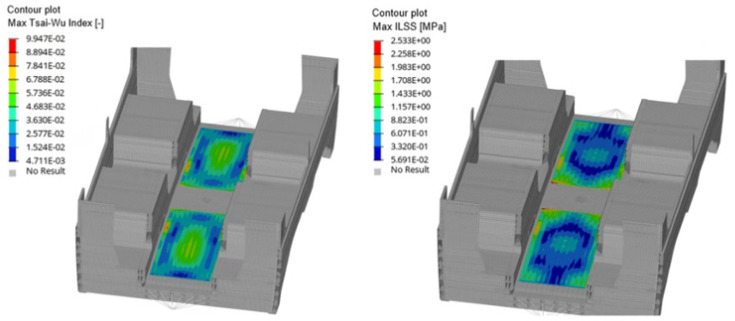
Final values distribution for the 36°/54°/126° configuration: max Tsai–Wu index, max ILSS.

The resulting data demonstrate a clear hierarchy in structural efficiency. The baseline configuration (0°), representing a standard non-optimized approach, exhibited the highest criticality with a Tsai–Wu index of 0.1560. The synchronous optimum (all plies at 54°) provided a significant improvement, reducing the failure index by 24.81%. This result confirms that the global principal stress path for the railway carbody component is oriented near the 54° axis. However, the most compelling result was achieved by the asynchronous strategy. The tailored layup 36°/54°/126° reached a failure index of 0.0923, yielding a total performance gain of 40.83% over the baseline. The additional 16% improvement of the asynchronous layup over the best synchronous case was a direct result of accounting for the stress tensor rotation through the laminate thickness. By allowing Ply 1 and Ply 3 to deviate from the global 54° mean (shifting to 36° and 126° respectively), the framework successfully “tuned” each layer to its local stress environment. This confirms that in complex railway structures, a uniform orientation is inherently sub-optimal, as internal layers are subjected to different loading conditions than the outer skins. Furthermore, while the optimized 36°/54°/126° sequence deviates from standard symmetry, the resulting bending–extension coupling effects remain negligible for this specific underframe geometry, ensuring structural stability without compromising the achieved mass reduction. A critical finding of this validation is the consistency of the interlaminar shear stress (ILSS). Across all simulations, the maximum ILSS remained stable at 2.53 MPa. This confirms that the aggressive optimization of in-plane angles did not compromise the interlaminar integrity of the laminate. Given that the values were well within the safe operating limits of the resin system, the 36°/54°/126° configuration was validated as both the most efficient and a safe design choice for the carbody component. Finally, Figure 12 illustrates the contour plots for the max Tsai–Wu index and the max ILSS across the carbody underframe. The numerical results demonstrate an absolute consistency between the two failure metrics, with distributions closely following the expected load paths. The most intense structural conditions were identified near the passenger seating areas, where the localized concentration of vertical loads, combined with the global bending of the underframe, leads to higher stress gradients. Despite these concentrations, both the Tsai–Wu and ILSS values remained well below the safety thresholds, confirming the robustness of the optimized laminate configuration under standardized operational loads

With reference to real test strategy, a multi-scale validation strategy should be used to verify the optimized configurations. At the material level, standardized coupon testing [56] should be performed to characterize the specific elastic constants and strength limits of the optimized asymmetric stacking sequences, ensuring that the numerical input data aligns with the manufactured ply behavior. At the component level, the strategy would involve testing on critical sub-assemblies, representing the main interaction between laminates and carbody frame. These tests should utilize strain gauge instrumentation and displacement transducers to correlate the local stiffness and onset of failure with the FEA predictions. Finally, modal testing on a representative prototype section should be conducted to validate the dynamic response, ensuring that the natural frequencies of the optimized lightweight structure remain within the safety margins required by railway regulations.

## 5. Conclusions

The research presented in this work focuses on the structural optimization of a composite laminate integrated within a railway carbody frame. The objective was to implement a systematic design methodology to achieve significant improvements in mechanical strength and failure resistance, as required by the stringent safety standards of the industry. The proposed framework enabled a complete evaluation of the laminate’s potential by exploring different stacking strategies and directly extracting performance data from high-fidelity finite element models. The core of the study initially focused on an asynchronous optimization strategy, where the orientation of each individual ply was decoupled and analyzed independently. This phase revealed that tailoring the fiber orientation to the specific position of the ply within the thickness is essential for maximizing performance. Through this ply-by-ply sensitivity analysis, the optimal sequence 36°/54°/126° was identified, achieving a 40.83% reduction in the Tsai–Wu failure index compared to the standard 0°/0°/0° baseline. Subsequently, a synchronous rotation analysis was conducted to evaluate the performance of the laminate when all plies are restricted to a single, uniform orientation. While this conventional approach identified an optimal global angle of 54°, it only yielded a 24.81% reduction in the failure index. The comparison between the two strategies clearly demonstrates the superiority of the asynchronous method, which provided an additional 16% performance gain by accounting for the varying structural requirements across the laminate layers. However, if a synchronous rotation was adopted for the sake of simplicity, aligning all plies with the principal load direction identified at 54°, the structural performance would still show significant improvement. This simplified configuration would benefit from the high efficiency of the internal layers, although it would lack the fine-tuned balancing achieved through the asynchronous method. In conclusion, the results confirm that the proposed methodology significantly enhances the structural safety margins of railway components. The optimization of in-plane orientations was achieved without compromising the interlaminar integrity, as the interlaminar shear stress (ILSS) remained constant at 2.53 MPa throughout the process. This systematic framework has been proven to be an effective and objective tool for lightweight design, replacing empirical trial-and-error methods with a rigorous performance-driven approach. Furthermore, the developed optimization strategy is material-agnostic; while this study focused on the high-performance requirements of CFRPs, the methodology can be readily applied to glass-fiber, hybrid, or bio-based composite systems in future industrial applications. This versatility ensures that the framework remains a robust and scalable solution for the evolving demands of sustainable, multi-material transport structures.

## Figures and Tables

**Table 1:** Mechanical properties of plies [53].

Ply	Material	Thickness [mm]	Orientation α [°]	Density [kg/m^3^]	E_1_ [GPa]	E_2_ [GPa]	ν_12_ [-]	G_12_ [GPa]	G_13_ [GPa]	G_23_ [GPa]
1	Carbon fiber/Epoxy	1	0	1590	135	8.0	0.30	5.0	5.0	3.0
2	Carbon fiber/Epoxy	1	0	1590	135	8.0	0.30	5.0	5.0	3.0
3	Carbon fiber/Epoxy	1	0	1590	135	8.0	0.30	5.0	5.0	3.0

**Table 2:** Plies’ failure parameters [53].

Material	Xt [MPa]	Xc [MPa]	Yt [MPa]	Yc [MPa]	S [MPa]	τ_il [MPa]
Carbon fiber/Epoxy	1500	800	60	160	80	60

**Table 3:** Final comparison results.

Strategy	Stacking Sequence	Max Tsai–Wu Index	Max ILSS [MPa]	Performance Gain
Baseline	0°/0°/0°	0.1560	2.53	-
Synchronous	54°/54°/54°	0.1173	2.53	+24.81%
Asynchronous	36°/54°/126°	0.0923	2.53	+40.83%

## Data Availability

The original contributions presented in the study are included in the article, further inquiries can be directed to the corresponding author.
